# Signaling lymphocytic activation molecule family receptors as potential immune therapeutic targets in solid tumors

**DOI:** 10.3389/fimmu.2024.1297473

**Published:** 2024-02-27

**Authors:** Metin Gunes, Steven T. Rosen, Idit Shachar, E. Gulsen Gunes

**Affiliations:** ^1^ Department of Hematology and Hematopoietic Cell Transplantation, Beckman Research Institute, City of Hope, Los Angeles, CA, United States; ^2^ Judy and Bernard Briskin Center for Multiple Myeloma Research, City of Hope, Los Angeles, CA, United States; ^3^ Department of System Immunology, Weizmann Institute of Science, Rehovot, Israel; ^4^ Toni Stephenson Lymphoma Center, City of Hope, Los Angeles, CA, United States

**Keywords:** signaling lymphocytic activation molecule family, SLAMF, cancer immunology, immunotherapy, solid tumors, tumor microenvironment

## Abstract

Recently, cancer immunotherapy has revolutionized cancer treatment. Various forms of immunotherapy have a manageable safety profile and result in prolongation of overall survival in patients with solid tumors, but only in a proportion of patients. Various factors in the tumor microenvironment play critical roles and may be responsible for this lack of therapeutic response. Signaling lymphocytic activation molecule family (SLAMF) members are increasingly being studied as factors impacting the tumor immune microenvironment. SLAMF members consist of nine receptors mainly expressed in immune cells. However, SLAMF receptors have also been detected in cancer cells, and they may be involved in a spectrum of anti-tumor immune responses. Here, we review the current knowledge of the expression of SLAMF receptors in solid tumors and tumor-infiltrating immune cells and their association with patient outcomes. Furthermore, we discuss the therapeutic potential of targeting SLAMF receptors to improve outcomes of cancer therapy in solid tumors. We believe the research on SLAMF receptor-targeted strategies may enhance anti-cancer immunity in patients with solid tumors and improve clinical outcomes.

## 1 Introduction

Cancer immunotherapy has revolutionized cancer treatment in the past decade, becoming the fourth pillar of treatment next to surgery, chemotherapy, and radiotherapy. Blocking immune checkpoints with monoclonal antibodies has improved outcomes in solid tumor patients (1). Furthermore, cellular therapies, particularly chimeric antigen receptor (CAR)-T cell therapy, have shown high effectiveness for various cancers (2). However, many patients with solid tumors do not benefit from these strategies. This has warranted research into resistance mechanisms and other treatment options. Some factors in the tumor microenvironment (TME) of solid tumors may contribute to resistance to immunotherapy (3). First, infiltration of cytotoxic lymphocytes is limited in many tumors (i.e., ‘cold’ tumors), which may be due to a lack of antigen presentation and recognition as well as physical and chemical barriers to infiltration. Furthermore, immunosuppressive TMEs, with infiltrates of suppressive immune populations that inhibit the anti-cancer immune response, may also limit the efficacy of cancer immunotherapies.

Signaling lymphocytic activation molecule family (SLAMF) receptors are increasingly being studied as potential factors that affect the immune environment in cancers and as potential targets for therapy. Numerous studies have overwhelmingly examined the structure and function of SLAMF receptors, their role in regulating the immune system, and possible strategies for targeting this receptor family therapeutically. However, our comprehension of the potential of SLAMF receptors in solid tumors is still incomplete. Our review highlights the potential of SLAMF receptors as targets for solid tumors and outlines their current targeting strategies.

## 2 SLAMF receptors

**Figure 1:**
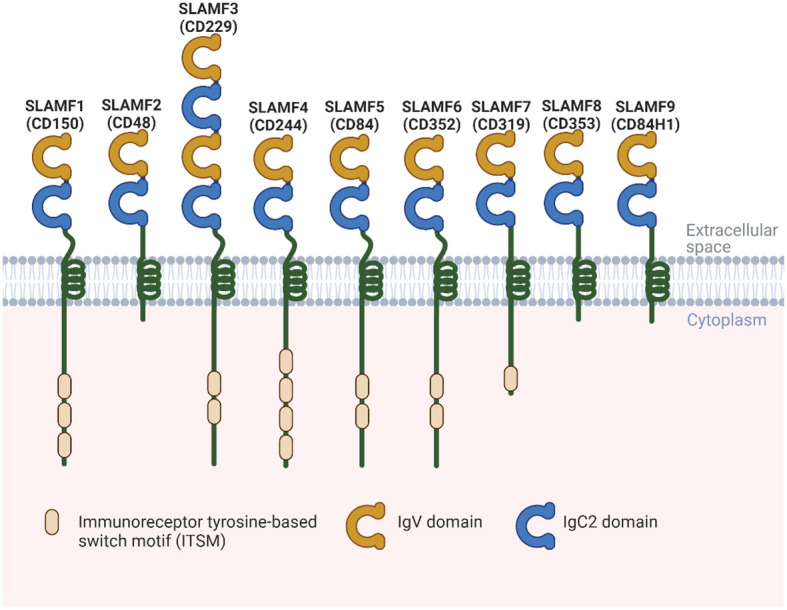
Structural representation of the human SLAMF receptors. SLAMF receptors are cell-surface receptors and are composed of nine members. They are type I glycoproteins that contain amino-terminal Ig-like variable domains (IgV) and membrane-proximal Ig-like constant two domains (IgC2) in their extracellular regions. The cytoplasmic region of every family member, except for SLAMF2, SLAMF8, and SLAMF9, contains ITSMs that mediate recruitment of SAP, as well as other SH2 domain-containing proteins such as EAT-2. Most of these receptors are homophilic, which can bind to one another, except for SLAMF2 and SLAMF4.

SLAMF receptors are a group of cell surface glycoproteins belonging to the immunoglobulin (Ig) superfamily of proteins involved in various immune functions. SLAMF consists of nine family members mostly expressed in immune cells. Most of these receptors are homophilic, except for SLAMF2 and SLAMF4, which can bind to one another (4). Each SLAMF receptor consists of an extracellular segment comprising two or four Ig-like domains, a transmembrane region, and a cytoplasmic tail. The cytoplasmic tails contain one or more copies of a tyrosine motif called immunoreceptor tyrosine-based switch motif (ITSM). However, SLAMF2, SLAMF8, and SLAMF9 lack most of the cytoplasmic tails (4) (**Figure 1**). When the receptors are engaged with their ligands, ITSMs get phosphorylated, which initiates interaction with intracellular SLAM-associated proteins, including SLAM-associated protein (SAP) and Ewing`s sarcoma-associated transcript 2 (EAT-2). These proteins contain an SH2 domain and serve as adaptor proteins to link SLAMF receptors to intracellular signaling pathways. When the N-terminal Ig domains of SLAMF receptors engage with their cognate ligands, these molecules are recruited, resulting in signaling transduction events that ultimately modulate various types of immune responses.

There is mounting evidence that SLAMF receptors and SAP-related adaptor molecules play essential and intricate roles in regulating the immune system. For instance, SAP adaptor molecules recruit Fyn, a Src family tyrosine kinase, leading to downstream phosphorylation and stimulation of activating signals within immune cells. SAP molecules also prevent recruitment of the SLAMF receptor to the inhibitory pathway mediated by SH2 domain-containing protein tyrosine phosphatase (SHP)-1, SHP-2, and SH2 domain-containing inositol phosphatase (SHIP)-1. In the absence of SAP adaptors, SLAMF receptors function as inhibitory signals in cellular activation. Similarly, EAT-2 functions by recruiting phospholipase C and preventing SLAMF receptors from coupling to inhibitory mediators. This enhances natural killer (NK) cell activity.

The importance of SLAMF receptors in the immune response became evident when the molecular defect responsible for X-Linked lymphoproliferative (XLP) syndrome was detected (5). The gene encodes SAP, and patients with this syndrome experience impaired immune responses. SLAMF receptors are known to be involved in NK- and T-cell development, expressed at various stages of B-cell development, and involved in B-cell regulation, antibody production, isotype switching, and NK-cell cytotoxicity. We have summarized the function of each SLAMF member and their expression on immune cells in **Table 1** (6–10).

**Table 1:** SLAMF receptors and their function and location on immune cells (6–10).

SLAMF Receptor	Expression and Function
**SLAMF1 (SLAM, CD150)**	Expressed on thymocytes, T cells, natural killer cells (NK), B cells, dendritic cells (DCs), macrophages, and hematopoietic stem cells (HSCs) and is involved in lymphocyte activation. In Crohn’s disease, an upregulation of SLAMF1 has been detected in monocytes and macrophages, and upregulation of SLAMF1 on T-cells was detected in rheumatoid arthritis. In contrast, in Chronic Lymphocytic Leukemia, it was found to be downregulated.
**SLAMF2 (CD48, BLAST1, BCM1)**	Expressed by NK cells, CD8^+^ T cells, B cells, ɣδ T cells, DCs, basophils, eosinophils, mast cells, and multipotent progenitor cells. SLAMF2 can bind CD2 as well as SLAMF4 to initiate signaling.
**SLAMF3 (CD229, LY9)**	Expressed on thymocytes, T cells, follicular helper T cells, B cells, DCs, macrophages, and NK cells. During antigen presentation by B cells, it is involved in creating the immunological synapse at the contact site between the T- and B cells.
**SLAMF4 (CD244, 2B4)**	Expressed on CD8^+^ T cells, ɣδ T cells, NK cells, DCs, macrophages, basophils, mast cells, and eosinophils. SLAMF4 binds SLAMF2, and this process is involved in NK-cell activation.
**SLAMF5 (CD84, LY9B)**	Expressed on thymocytes, T cells, follicular helper T cells, B cells, NK cells, macrophages, DCs, basophils, mast cells, eosinophils, and platelets. Its signaling can stimulate platelets and is involved in T-cell activation, resulting in IFNγ production.
**SLAMF6 (CD352, NTBA, LY108)**	Expression can be found on thymocytes, T cells, B cells, NK cells, DCs, neutrophils, and eosinophils. It has been found to be involved in NK-cell cytotoxicity and cytokine production, T-cell activation, and neutrophil functions.
**SLAMF7 (CD319, CS1, CRACC)**	Expressed by T cells, B cells, NK cells, NKT cells, DCs, and macrophages and has been shown to regulate NK-cell cytolysis and can partially rescue effector functions in NK- and CD8^+^ T cells.
**SLAMF8 (CD353, BLAME, SBBI42)**	Expression detected on macrophages and faintly expressed on B-cell subsets.
**SLAMF9 (CD2F10, CD84H1)**	Expression was detected on T cells, B cells, NK cells, and DCs. It is the most recently described SLAMF member, and its ligand has not yet been discovered.

**Figure 2:**
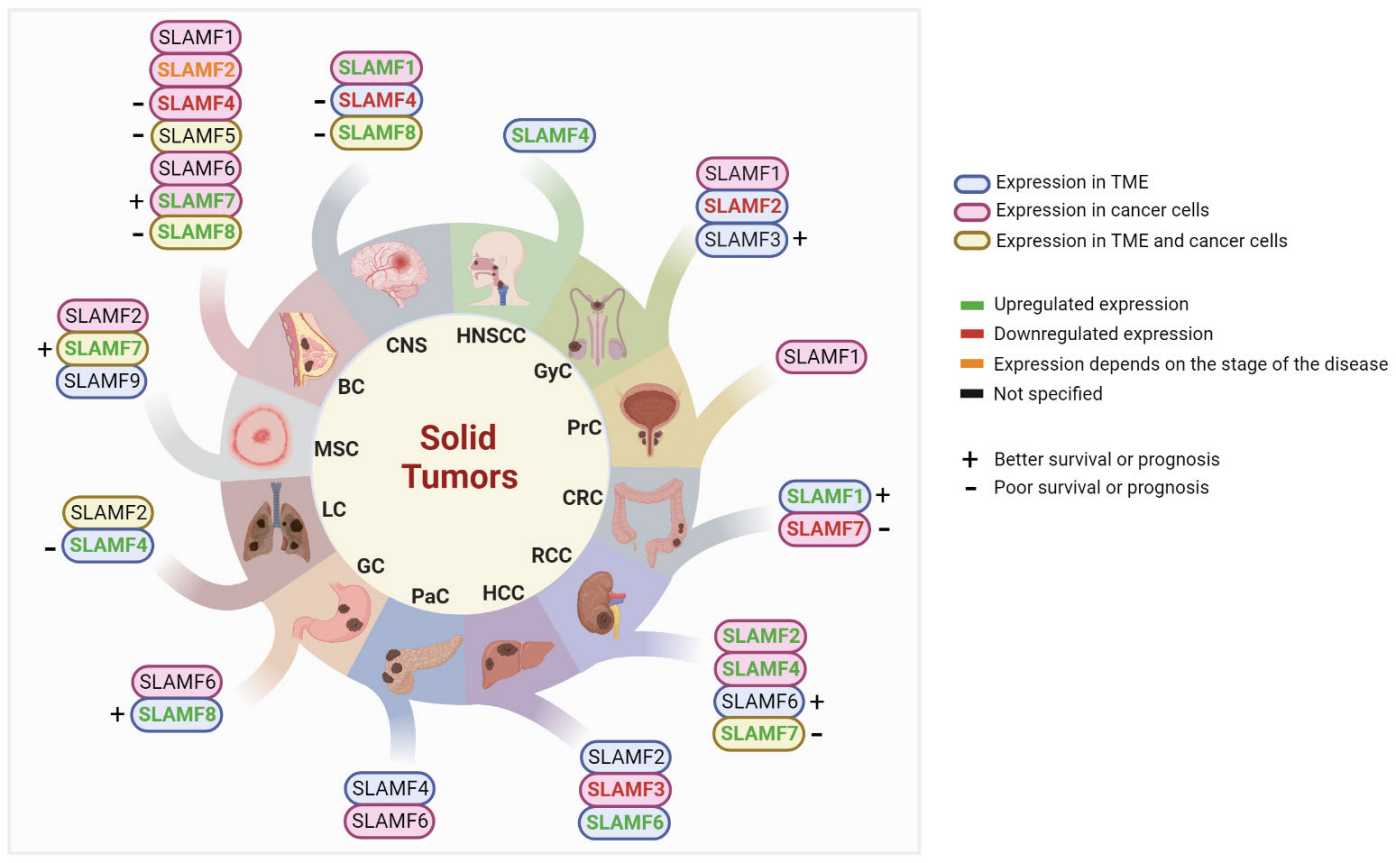
Studied SLAMF receptors in solid tumors. BC, breast cancer; CNS, central nervous system; CRC, colorectal cancer; GC, gastric cancer; GyC, gynecological cancer; HCC, hepatocellular carcinoma; HNSCC, head and neck squamous cell carcinoma; LC, lung cancer; MSC, melanoma skin cancer; PaC, pancreatic cancer; PrC, Prostate cancer; RCC, renal cell carcinoma; TME, tumor microenvironment.

With recent research, the role of SLAMF receptors in solid tumors and the immune response against these tumors has become more evident. For instance, the upregulation of various dendritic cell (DCs) markers, including CD80, CD274, and SLAMF1, was associated with improved overall survival (OS) in a mixed cancer analysis (11). Here, we will describe the current data on SLAMF receptor expression in solid tumor types (**Figure 2**), potential associations with prognosis and therapy response, and potential targeted therapy strategies. Of note, the order of discussion will start with the solid tumor types that have more data available in the literature.

## 3 SLAMF receptors in solid tumors

### 3.1 Breast cancer

Several investigations have shown the variable expression of multiple SLAMF members in breast cancer. SLAMF1/CD150 was not found to be expressed on the cell surface of breast cancer cell lines. However, it was detected in the cytoplasm of 45% of cell lines. The highest expression levels were detected in cell lines representing a luminal subtype (T47D), while basal-type cell lines, such as MDA-MB-231, BCC/P, and BC/ML, expressed lower levels. Additionally, cell lines expressed variable levels of mRNA encoding the transmembrane mCD150 and the so-called novel CD150 (nCD150) isoforms. Assessment of public databases with patient DNA microarray data also showed that breast tumors express SLAMF1 (12). Furthermore, it was found that the SLAMF1 single nucleotide polymorphism (SNP) rs1061217 was associated with a decreased risk of breast cancer in overweight women, while it increased the risk of breast cancer in those with normal weight (13).

SLAMF2/CD48 has not been studied extensively in breast cancer. An analysis of the expression of NF-kappa B (NF-κB) related genes using RT-PCR in inflammatory breast cancer revealed that *CD48* was upregulated in these samples compared to invasive ductal carcinomas. When comparing biopsies of distant metastases of non-inflammatory breast cancer, *CD48* was one of six downregulated genes compared to the primary invasive ductal carcinomas (14).

In a large analysis of immune checkpoint genes in breast cancer, SLAMF4/CD244 expression in tumors was found to be lower than that in healthy breast tissue (15). In another study, a gene analysis in triple-negative breast cancer (TNBC) showed that overexpression of *Prune-1*, *IL-10*, *COL4A1*, *ILR1*, and *PDGFB*, as well as inactivating mutations of *PDE9A*, *CD244*, *Sirpb1b*, *SV140*, *Iqca1*, and *PIP5K1B* genes, are associated with metastasis to the lungs, suggesting low expression of CD244 may be associated with worse prognosis. This was confirmed in a The Cancer Genome Atlas (TCGA) analysis, which showed that low expression of the CD244 gene was associated with decreased survival (16). Additionally, in a BRCA2-deficient breast cancer mouse model, missense mutations in the *CD244* receptor domain were detected (17). These data suggest that a loss of CD244 signaling may contribute to a worse prognosis in breast cancer.

SLAMF5/CD84 was detected as an identifying marker for myeloid-derived suppressor cells (MDSCs) in breast cancer in a mouse model, and *in vitro* experiments showed that PBMC-derived human MDSCs upregulate SLAMF5. Co-culture experiments with such CD84^hi^ MDSC showed that they actively inhibit T-cell proliferation (18). In TCGA, *CD84* was found to be an independent negative prognostic factor for both disease-free survival (DFS) and OS. Furthermore, in circulating tumor cells, *CD84* expression was associated with a mesenchymal phenotype (19).

Another TCGA analysis that assessed core genes associated with breast cancer status revealed SLAMF6/CD352 as one of eight core genes. However, SLAMF6 had only a weak association with survival (P=0.042) and no significant association with tumor (T), node (N), and metastases (M) (TNM) status and was not further assessed (20).

SLAMF7/CD319 mRNA expression was found to be enriched in breast cancer TCGA analysis, as compared to healthy breast tissue (21, 22). A study in lymph node-positive breast cancer of various subtypes showed moderate or strong protein expression of SLAMF7 in the cytoplasm in approximately 20% of cases, while 80% had no or weak expression. In samples with high expression, up to 70% of tumor cells expressed high levels of SLAMF7. Higher levels were associated with younger age, less evasive tumors, and better prognosis. Patients with high expression had a lower relapse rate and longer disease-specific survival (DSS). However, multivariate analysis did not show SLAMF7 as an independent prognostic factor. The researchers also detected a weak association between highly vascular invasive cells and low expression levels (P=0.05) (21).

In addition, one study found a correlation between a high expression of SLAMF8/CD353, tumor necrosis factor (TNF), and lymphocyte infiltration with a poor response to therapy in postmenopausal estrogen receptor (ER)^+^ breast cancer (23).

### 3.2 Central nervous system tumors

While SLAMF1 is not found in healthy brain tissue, 77.6% of the human central nervous system (CNS) tumors were found to express it. These tumors included glioblastoma, anaplastic astrocytoma, diffuse astrocytoma, and ependymoma. SLAMF1 was detected only in the cytoplasm of tumor cells. The novel CD150 (nCD150) transcript was also detected at high levels in these tumors, and this isoform was the predominant form in glioma cells (24).

In patients with glioblastoma, blood plasma was analyzed for the expression of various proteins that may be associated with prognosis. Low plasma levels of SLAMF4 were associated with short progression-free survival (PFS) (25).

In an assessment of TCGA glioma and the Chinese Glioma Genomic Atlas (CGGA) data, overexpression of SLAMF8 was associated with progression, higher grade glioma, and it was a biomarker for the mesenchymal subtype. The highest levels of SLAMF8 were found in glioblastoma, and in this cancer type, it was associated with reduced OS and chemoresistance. The overexpression of SLAMF8 was associated with higher infiltration of monocytes, myeloid DCs, and fibroblasts and with genes related to acute and chronic inflammation. Furthermore, it was strongly correlated with the expression of checkpoint molecules CTLA-4, PD-1, PD-L2, B7-H3, and TIM-3, but not PD-L1 (26). These data suggest SLAMF8 may be implicated in an immunosuppressive tumor microenvironment.

Besides CNS tumors, SLAMF proteins have also been implicated in stroke. Mice lacking SLAMF5 on platelets or T cells had reduced cerebral infiltration of CD4^+^ T-cells and reduced thrombolytic activity after experimental stroke, resulting in a reduction of neurological damage. Furthermore, human arterial blood samples from the ischemic cerebral circulation showed local shedding of SLAMF5, and high expression of CD84 on platelets was associated with poor outcomes in patients with stroke (27).

### 3.3 Lung cancer

Non-small cell lung cancer (NSCLC) is relatively resistant to NK-cell-mediated cytotoxicity. Park et al. assessed various cell lines with variable sensitivity to NK-cell killing and found that SLAMF2 expression made the cells susceptible to killing. SLAMF2 increased the stability of the contact between the cancer cells and NK cells in live imaging experiments, which might explain this killing relationship (28).

In a lung cancer model with sepsis, PD-1 checkpoint inhibition has no effect on sepsis survival. SLAMF4 was found to be a checkpoint of interest in this condition, and the blockade of SLAMF4 improved sepsis survival. It was associated with T-cell costimulatory receptor expression and decreased coinhibitory receptor expression (29). In patients with stage I NSCLC, blood levels of SLAMF4 were found to be a prognostic factor, and those with high levels of SLAMF4 had worse PFS. This study suggested that the expression of SLAMF4 was mainly found on the immune infiltrate (30). This was confirmed in a mouse model with subcutaneous lung cancer. In these tumors, the frequencies of PD1^+^, BTLA^+^, and SLAMF4^+^ CD4^+^ and CD8^+^ T-cells were increased, and CD8+ T-cells expressing SLAMF4 produced reduced levels of IL-2 and IFNγ (31). Therefore, the blockade of SLAMF4 might be of interest for the therapy of lung tumors.

SLAMF5 has been found to play a role in other lung diseases. In a mouse model for mycobacterium tuberculosis infection, levels of SLAMF5 increase on T- and B-cells in the lung tissue of infected mice, which is also seen in peripheral blood mononuclear cells (PBMCs) of patients with pulmonary tuberculosis. This expression resulted in immunosuppression, inhibiting T- and B-cell activation (32). SLAMF5 may, therefore, serve as a target for therapy in this disease, as well as in lung cancers, due to its potential on the immune cells of the lung tissue.

### 3.4 Pancreatic cancer

In a mouse model of pancreatic cancer infected with Listeria monocytogenes, bacteria antigen-specific CD8^+^ and total T cells had increased expression of BTLA, PD-1, and SLAMF4. Expression of these markers reduced IFNγ and increased IL-2 production of CD8^+^ T-cells. These data suggest that suppressive effects in the TME might also affect immune responses to bacterial infections (33).

In a screening of genes associated with OS and DFS in pancreatic ductal adenocarcinoma (PDAC), a 7-gene signature containing SLAMF6 was found to be associated with survival. This suggests SLAMF6 might play an interesting role in pancreatic cancer, but further research would be required to study the role it plays within this gene signature (34).

### 3.5 Prostate cancer

SLAMF1 cell surface and cytoplasmic expression have been detected in the prostate cancer cell lines LNCap, Du-145, and PC-3. The highest expression levels were found in the less aggressive androgen therapy-responsive non-metastatic LNCap cells. The cell lines also expressed novel nCD150 isoforms, and soluble CD150 was detected at low levels for the LNCap and PC-3 cell lines (12). Whether the expression of SLAMF1 is associated with clinical outcomes in prostate cancer remains to be determined.

### 3.6 Gastric cancer

SLAMF receptors have been implicated in some studies of gastric cancer. An analysis comparing cancerous with non-cancerous tissue found genes that could predict survival, and SLAM was one of the genes (35).

Circular RNA is a form of non-coding RNA, and circSLAMF6 can be generated from back splicing of the SLAMF6 first intron. In hypoxic conditions, circular RNA SLAMF6 (circSLAMF6) is increased in gastric cancer cells *in vitro*. This increase is associated with glycolysis, migration, and invasion of these tumor cells, and the knockdown of circSLAMF6 reverses these effects. In a mouse model for gastric cancer, circSLAMF6 deficiency inhibited tumor growth by regulating the miR-204-5p/MYH9 axis (36).

High levels of SLAMF8 have been detected in the serum of patients with gastric cancer (37). Furthermore, investigations in a gastric cancer model with Epstein-Barr virus (EBV) infection, which has been associated with improved responses to anti-PD-1 therapy, high SLAMF8 expression was found to be a factor that might be involved in these responses. High expression of SLAMF8 was associated with T-cell activation gene enrichment, CD8 expression, and better response to anti-PD-1 checkpoint blockade therapy. SLAMF8 in this setting was mostly expressed by macrophages, and overexpression of SLAMF8 in macrophages resulted in gene enrichment of multiple immune-related pathways. Therefore, SLAMF8 is correlated with immune ‘hot’ gastric cancers that respond better to immune checkpoint blockade (38).

### 3.7 Colorectal cancer

Research in CRC has suggested that SLAMF1 and SLAMF7 may be of interest. Transfection of CD3-activated T-cells with SLAMF1 increased their cytotoxic activity and IFNγ production *in vitro* against human colon cancer cells. In xenograft models, these T cells reduced tumor growth, suggesting increased SLAMF1 expression on T cells in colon cancer may be beneficial (39). In the human CRC TME, SLAMF1 was detected on tumor-specific innate lymphoid cells, and these cells were observed at higher levels in patient blood than in healthy controls. Patients with high levels of SLAMF1 expression had a better survival rate than those with low expression, suggesting SLAMF1 to be a marker for improved anti-tumor activity (40). Additionally, SLAMF1 was detected as one of four core genes impacting prognosis in colon adenocarcinoma in an investigation into immune-related subtypes from TCGA (41).

SLAMF7 has been found to be downregulated in CRC tissue as compared to healthy tissue. In CRC cells overexpressing SLAMF7, CD68, and CD73 were downregulated after co-culture with a monocytic cell line, suggesting SLAMF7 might play a role in suppressing these markers (42). In another study, SLAMF7 expression did not differ between paracancer and tumor tissue or correlate with the TNM stage. In patients treated with chemoimmunotherapy and adjuvant immunotherapy based on cytokine-induced killer cells combined with chemotherapy, no correlation was found between SLAMF7 expression and CD8^+^ T-cell or NK-cell infiltration. However, a higher expression of SLAMF7 was associated with better OS (43). Therefore, the role of SLAMF7 in CRC and its relationship to the immune response requires further investigation.

### 3.8 Hepatocellular carcinoma

In HCC, the number of activated, functional NK cells is associated with improved outcomes. In advanced HCC, fewer of these NK cells are detected, and the cells present have impaired TNFα and IFNγ production, suggesting limited functionality. This was shown to be associated with high infiltration of peritumoral stroma monocytes and macrophages. *In vitro*, NK cells exposed to these monocytes could undergo a rapid transient activation, resulting in exhaustion and, eventually, cell death, suggesting this might be the reason for limited NK-cell function in advanced HCC. The mechanisms behind this interaction might be associated with SLAMF signaling. Monocytes in HCC express high levels of SLAMF2, and *in vitro* experiments showed that the effects of monocytes on NK cells could be reduced by blocking SLAMF4 on the NK cells, suggesting a direct role of SLAMF2-4 signaling in these NK-cell exhaustion effects (44).

Healthy hepatocytes have been shown to express SLAMF3, but no other SLAMF members. In primary HCC samples, resected tumor samples, and HCC cell lines, the expression of SLAMF3 was significantly lower than in healthy cells, suggesting downregulation when hepatocytes undergo tumorigenesis. Restoration of high levels of SLAMF3 in cell lines was shown to inhibit cell proliferation and migration and enhance apoptosis. Additionally, these cells progressed less in nude mice than in their low SLAMF3 counterparts. Mechanistically, SLAMF3 may be associated with the signaling of various pathways, as expression resulted in decreased phosphorylation of MAPK, ERK 1/2, JNK, and mTOR (45). Follow-up studies showed that the inhibitory effect of SLAMF3 on HCC proliferation occurs through a retinoblastoma (RB) factor and PLK1-dependent pathway. Expression of SLAMF3 retained RB factor in its hypophosphorylated active form, which inactivates the transcription factor E2F, and represses the expression and activation of PLK1. PLK1 is a cell cycle protein that promotes cell cycle progression. In human samples, this was confirmed with an inverse correlation between SLAMF3 and PLK expression (46). Additionally, induction of SLAMF3 was associated with loss of MRP-1 expression, a drug resistance transporter. In patient samples, an inverse correlation between SLAMF3 and MRP-1 expression was also detected, suggesting that loss of SLAMF3 expression in tumor cells may be associated with drug resistance (47).

SLAMF6 levels were found to be increased on CD14^+^ cells derived from blood from patients with HCC, which was associated with positive Hep B virus DNA status and high levels of α-fetoprotein. *In vitro* and *in vivo* experiments in mice showed that tumor-associated macrophages (TAMs) had higher levels of SLAMF6 (Ly108), and this was associated with the M2 phenotype. Small interfering RNA blocking Ly108 resulted in suppression of M2 macrophage polarization. Macrophages with suppressed SLAMF6 levels were able to reduce HCC cell migration and invasion and could prevent tumor growth. This latter effect was associated with the inhibition of the NF-κB pathway in macrophages, which plays a role in macrophage polarization (48).

### 3.9 Melanoma skin cancer

Several SLAMF members have been implicated in melanoma. In a murine model, inoculation with SLAMF2^+^ and SLAMF2^-^ metastatic B16 melanoma cells showed that WT mice had trouble rejecting the SLAMF2^+^ tumors compared to SLAMF2^-^ melanoma cells. In mice lacking SLAMF4, there was a difference between the rejection rates of these cells in male and female mice. Male mice lacking SLAMF4 rejected SLAMF2^+^ melanoma cells, while female mice lacking SLAMF4 had trouble rejecting both SLAMF2^+^ and SLAMF2^-^ cells. These gender-specific differences might be related to differences in NK-cell function (49).

Eisenberg et al. created a 203-amino acid sequence of the human SLAMF6 (seSLAMF6) ectodomain. This molecule reduced activation-induced cell death in tumor-infiltrating lymphocytes (TIL). When CD8^+^ T-cells were costimulated with seSLAMF6, the cells secreted more IFNγ and had improved cytolytic activity. When these cells were injected into the B16F10 melanoma mouse model, it delayed tumor growth, which could be further enhanced by treating the mice with seSLAMF6 (50). Another study showed that inhibition of SLAMF6 with an anti-SLAMF6 antibody affected tumor growth of the B16 melanoma model. Exhausted CD8^+^ T-cells had increased degranulation when anti-SLAMF6 was added to the culture (51). Similar results were obtained when SLAMF6-negative Pmel-1 cells specific for gp100 were created. Upon activation, these cells acquired an effector memory phenotype and showed improved polyfunctionality and strong tumor cytolysis. Adoptive transfer of these cells into mice-bearing melanoma tumors resulted in lasting tumor regression. Given that the CD8^+^ T-cells in this model expressed high levels of LAG3, adding anti-LAG3 checkpoint blockade could further improve anti-tumor responses (52).

TCGA analysis has revealed an enrichment of SLAMF7 in melanoma and a correlation between SLAMF7 and favorable prognosis. The expression of SLAMF7 was negatively correlated with NK-cell markers, suggesting that the expression of SLAMF7 in these tumors is unlikely NK-cell expression. *In vitro* studies showed that agonistic engagement of SLAMF7 on tumor-specific CD4^+^ T-cells enhanced their cytolytic activity, which, if expressed by CD4^+^ T-cells in these tumors, may explain the relationship with favorable prognosis (22).

Finally, SLAMF9-expressing TAMs have been detected in 73.3% of human melanomas, 95.5% of naevi of melanoma patients, and 50% of naevi of healthy controls. SLAMF9 was also expressed in melanocytes in 20% of melanoma samples and 2.3% of naevi from melanoma patients but not in healthy controls. *In vitro* experiments showed that SLAMF9 gene expression was upregulated in murine bone marrow-derived macrophages stimulated with tumor-conditioned media of B16F10 melanoma cells. Furthermore, SLAMF9 expression enhanced TNFα secretion after LPS stimulation, and it delayed wound closure of RAW 264.7 cells in a scratch assay (53).

### 3.10 Renal cell carcinoma

A TCGA analysis into immune checkpoints in clear cell RCC (ccRCC) revealed that although these receptor/ligands were not found to be the most relevant in this study, genes encoding SLAMF2 and SLAMF4 were found to be more highly expressed in tumor tissue as compared to adjacent non-tumor tissue (54).

An analysis in ccRCC focused on regulatory T cells (Tregs) in tumor tissue and found that SLAMF6 is one of four hub genes related to prognosis and Tregs and associated with a better outcome (55).

Another TCGA analysis showed that SLAMF7 strongly correlated with various inhibitory receptors and that high expression was correlated with poor survival in ccRCC. CyTOF analysis of the TME of 73 ccRCC patients revealed that SLAMF7 was expressed by TAMs, with a unique subset of SLAMF7^hi^CD38^hi^ TAMs; these cells correlated with exhausted T-cells and were an independent prognostic factor. In co-culture experiments, it was shown that SLAMF7-SLAMF7 interactions between murine TAMs and CD8^+^ T-cells induced the expression of inhibitory receptors. In mice lacking SLAMF7, B16F10 growth was restricted, and CD8^+^ T-cells in these tumors expressed lower levels of PD-1 and TOX, suggesting a less exhausted phenotype (56).

### 3.11 Gynecological cancers

SLAMF1 was found to activate autophagy-related mechanisms that promoted resistance to methotrexate in choriocarcinoma cells. Depletion of SLAMF1 suppressed autophagy and induced apoptosis of MTX-resistant cell lines, which overexpressed SLAMF1 (57, 58).

Choriocarcinoma cells can be resistant to NK-cell lysis. This was associated with a lack of NK-cell activation, as choriocarcinoma cells lacked expression of SLAMF2, the ligand for SLAMF4 (59).

Limited research is available on SLAMF expression in ovarian cancer. Assessment of TCGA and University of California, Santa Cruz (UCSC) ovarian cancer datasets revealed that various SLAMF members were part of a hub gene profile in immune infiltrates. This hub gene profile included SLAMF1, SLAMF3, SLAMF6, and SLAMF7. Two of these, SLAMF1 and SLAMF3, were recognized as the real hub genes in immune infiltrates in ovarian cancer. These genes were associated with OS, which was related to their effect on the infiltration of activated B-cells (60). Therefore, these SLAMF members may be of interest for immunotherapy for ovarian cancer.

Attempting to construct a BRCAness signature for ovarian cancer, Chen et al. found that upregulation of CXCL1 with downregulation of SV2A and upregulation of SLAMF3 with downregulation of CHRNB3 can be constructed as a two-gene pair signature for BRCAness in ovarian cancer that predicts improved OS, PFS, and increased multi-omics alterations in homologous recombination genes. Furthermore, these could predict enhanced sensitivity to immune checkpoint blockade and poly ADP ribose polymerase (PARP) inhibitors, confirming SLAMF3 as an attractive immunotherapeutic target in ovarian cancer (61).

### 3.12 Head and neck squamous cell carcinoma

CD8^+^ TIL in HNSCC tumors has been found to express increased levels of SLAMF4, and this expression was correlated with PD-1 expression. Furthermore, SLAMF4 was increased on intratumoral DC and MDSC, and high SLAMF4 correlated with PD-L1 expression and increased expression of immune-suppressive mediators. *In vitro* studies showed that activation of SLAMF4 inhibited the production of pro-inflammatory cytokines by human DCs. CD244^-/-^ mice showed impaired tumor growth of HNSCC, and anti-SLAMF4 treatment also impaired the growth of established HNSCC tumors while it increased CD8^+^ TIL infiltration, suggesting SLAMF4 plays an inhibitory role in the immune response to HNSCC (62).

We have summarized the described expression and roles of SLAMF members in the TME of solid tumors in **Table 2**.

**Table 2:** Described expression and roles of SLAMF members in the TME of solid tumors.

SLAMF Receptor	Expression in Cancer	Role in Cancer	Tumor Microenvironment	References
**SLAMF1 (CD150, SLAM)**	• BC (incl. novel CD150) • CNS tumors (incl. novel CD150) • PrC • GyC • CRC	• Expressed in breast cancer cell lines, with high levels on those of the luminal type • Highest expression detected in aggressive prostate cancer cell lines • Drives autophagy and chemotherapy resistance in choriocarcinoma	• Expression on immune cells (T cells, B cells, and innate lymphoid cells) in CRC – associated with survival	(12, 24, 39–41, 56, 57, 59)
**SLAMF2 (CD48, BLAST1, BCM1)**	• BC • LC • MSC • ccRCC • HCC • GyC	• Upregulated in inflammatory breast cancer, downregulated in breast cancer metastases • Expression in lung cancer cell lines increases susceptibility to NK-cell killing • Expression in mouse melanoma tumors reduces tumor rejection • Upregulated in ccRCC	• Monocytes in HCC express high levels • SLAMF2 expression in NSCLC cells increases susceptibility to NK-cell killing • Lack of SLAMF2 associated with resistance to NK-cell lysis in choriocarcinoma cells	(14, 28, 44, 49, 53, 58)
**SLAMF3 (CD229, LY9)**	• GyC • HCC	• Loss of SLAMF3 in HCC might be associated with drug resistance	• Expressed on immune infiltrate (B cells) in ovarian cancer	(45, 47, 59, 60)
**SLAMF4 (CD244, 2B4)**	• HNSCC • NSCLC • ccRCC • PaC • BC • CNS	• Reduced expression in breast cancer associated with worse prognosis • Low levels in blood plasma in glioblastoma are associated with poor outcome • In NSCLC, high blood levels predict worse outcomes • Upregulated in ccRCC	• Expressed on immune cells in NSCLC • Expression on T cells in pancreatic cancer mouse model • Expressed on CD8^+^ TIL, DCs, and MDSCs in HNSCC	(15, 16, 25, 30, 33, 61)
**SLAMF5 (CD84, LY9B)**	• BC	• Expression on circulating tumor cells of mesenchymal breast cancer	• Expressed by MDSC in breast cancer – correlated with worse outcomes	(18, 19)
**SLAMF6 (CD352, NTBA, LY108)**	• BC • PDAC • GC (circular SLAMF6) • RCC • HCC	• Weak association with survival in breast cancer • Potential role in PDAC outcomes • Gastric cancer mouse models: circular SLAMF6 in hypoxia associated with more aggressive subtypes	• Associated with Tregs in RCC – associated with improved outcomes • Increased on CD14^+^ cells in HCC (M2 TAMs)	(20, 34, 36, 48, 54)
**SLAMF7 (CD319, CS1, CRACC)**	• BC CRC* • ccRCC • MSC	• Enriched in breast cancer – associated with better prognosis Downregulated in CRC* • Enriched in melanoma – associated with improved outcome • High expression in ccRCC is associated with worse outcome	• Expression in melanoma might be associated with CD4^+^ T-cell expression • Expressed by TAMs in ccRCC	(21, 22, 42, 55)
**SLAMF8 (CD353, BLAME, SBBI42)**	• BC • CNS • GC (serum)	• High expression in breast cancer associated with poor therapy response • Overexpression in glioma associated with disease progression, poor survival, and chemoresistance • Associated with better response to checkpoint inhibitors when expressed in serum gastric cancer	• Expression in breast cancer associated with TNF and lymphocyte infiltration • Associated with infiltration of myeloid cells, correlated with checkpoint expression in glioma. • Expressed by macrophages in gastric cancer	(23, 26, 37, 38)
**SLAMF9 (CD2F10, CD84H1)**	• MSC		• Expressed on TAMs in melanoma	(52)

BC, breast cancer; ccRCC, clear cell renal cell carcinoma; CNS, central nervous system; CRC, colorectal cancer; DC, dendritic cell; GC, gastric cancer; GyC, gynecological cancer; HCC, hepatocellular carcinoma; HNSCC, head and neck squamous cell carcinoma; LC, lung cancer; MSC, melanoma skin cancer; NK, natural killer; NSCLC, non-small cell lung cancer; MDSC, monocyte-derived suppressor cell; PaC, pancreatic cancer; PDAC, pancreatic ductal adenocarcinoma; PrC, Prostate cancer; RCC, renal cell carcinoma; SLAMF, signaling lymphocytic activation molecule family; TAM, tumor-associated macrophages; TIL, tumor-infiltrating lymphocyte; TNF, tumor necrosis factor.

## 4 Conclusion and future directions

Ample evidence suggests that SLAMF receptors are involved in in various solid tumor types is coming to light, suggesting that these receptors might be potential targets for therapy. SLAMF1 has been detected in various cancer types, but its role in prognosis remains to be established. However, the expression of SLAMF1 on immune cells in tumors might benefit the outcome. SLAMF2 and SLAMF4 have mainly been detected on T- and NK cells in tumors and may affect the ability of the immune system to control solid tumors. On the other hand, SLAM-family receptors, particularly SLAMF4, may be inhibitory or activatory in cells with SAP adaptor molecules, depending on the situation (63). Research on SLAMF3 remains limited. In a recent study, it has been reported that SLAMF3 stimulates the differentiation of Th17 cells from CD4+ T cells, leading to an increase in the secretion of IL-17A in a chronic (long-lasting) autoimmune disorder (64). However, in solid tumors, the expression loss on hepatic cancer cells is associated with poor outcomes, and expression on immune cells in ovarian cancer potentially results in better outcomes. SLAMF5 has also had limited investigation in solid tumors, but its detection on MDSC in breast cancer suggests it might be a therapeutic target of interest. SLAMF6 appears to be associated with outcomes in various cancer types and is expressed by various immune cells, with variable anti-tumor effects.

SLAMF7 has been found enriched in various solid tumor types, which may be associated with CD4^+^ T-cells and TAMs expression. According to the researchers, the process of differentiation from monocytes to macrophages results in increased expression of SLAMF7. This up-regulation of SLAMF7 promotes the induction of cytokines by certain Toll-like receptor ligands, suggesting that the differentiation of macrophages in solid tumors might involve a pathway through SLAMF7 (65). SLAMF7 has also been shown to be effective in improving survival when combined with lenalidomide and dexamethasone with the monoclonal antibody elotuzumab in patients with multiple myeloma (66). The use of this antibody in the clinic paves the way for research into the effects of this treatment in tumor types overexpressing SLAMF7. However, given that current research shows potentially improved outcomes with high expression, the mechanism of action will be important to explore. SLAMF8 expression in tumors was associated with worse outcomes in breast cancer and glioma, while serum expression in gastric cancer was associated with a good response to immunotherapy. SLAMF9 has not been assessed in great detail in the solid cancer setting, but research showing expression on TAMs in melanoma suggests it might be a target for further research.

In this review, we have specifically discussed increasing evidence of the roles of SLAMF receptors in various solid tumors that may improve patient outcomes. We have also suggested several ways to target SLAMF receptors in solid tumors. Together, these data suggest that SLAMF members play variable roles in solid tumors. While research should be expanded to uncover their roles in prognosis and expression patterns on various cells in the TME, an argument can be made to investigate these molecules for therapeutic purposes. However, targeting SLAMF receptors could also impact the normal immune response and increase the risk of infections due to their complex regulatory functions within the immune system (67). Therefore, it is crucial to take into consideration the potential risks associated with targeting SLAMF receptors and to take appropriate safety measures to minimize the potential toxicities, such as neutropenia, thrombocytopenia, and hepatotoxicity (63).

## Author contributions

MG: Data curation, Investigation, Writing – original draft, Writing – review & editing. SR: Supervision, Writing – review & editing. IS: Supervision, Writing – review & editing. EG: Conceptualization, Data curation, Investigation, Writing – original draft, Writing – review & editing, Supervision.
